# Non-IgE-mediated food allergy: a tolerance induction protocol

**DOI:** 10.1186/2045-7022-3-S3-P19

**Published:** 2013-07-25

**Authors:** C Arêde, A Cabral, S Piedade, M Morais-Almeida

**Affiliations:** 1 Immunoallergy Department, CUF Descobertas Hospital, Lisbon, Portugal

## Background

Cow’s milk proteins allergy (CMPA) is the most common food allergy in childhood, affecting up to 2.5% of infants. The immune mechanisms underlying non-IgE-mediated clinical presentations, which mostly affect the gastrointestinal system and are sometimes very severe, are not fully understood. Currently, specific oral tolerance induction (SOTI) is available as a specific treatment for CMPA, but most studies of this approach have focused on IgE-mediated forms of this food allergy.

## Methods

Children, with non-IgE-mediated CMPA, underwent SOTI using a protocol consisting of oral ingestion of increasing doses of cow’s milk, always in Hospital settings, until reaching a 200 mL dose per day, before introduction of an unrestricted diet.

## Results

Five children aged from 19 months to 4 years (3 males, 2 females) with a history of CMPA, since the first year of life and predominantly manifested by gastrointestinal symptoms, were included. All patients have completed the protocol and remain on an unrestricted diet. During SOTI three children had mild to moderate reactions, mainly abdominal pain and with spontaneous resolution, but sometimes justifying protocol adjustment.

## Conclusion

Despite clinical and immunological differences between IgE and non IgE CMPA, this protocol appears also to be a safe and effective alternative in the management of non-IgE-mediated CMPA, reducing the risk of accidental reactions and improving quality of life of chidren and their families. Nevertheless further studies are needed to validate this procedure.

## Disclosure of interest

None declared.

